# Supplementary material for: Causal Relationship between Aging and Anorexia Nervosa: A White-Matter-Microstructure-Mediated Mendelian Randomization Analysis
Source: Biomedicines. 2024 Aug 16;12(8):1874. doi: 10.3390/biomedicines12081874 (PMC11351342; doi:10.3390/biomedicines12081874)
Supplement: Supplementary file 1 [file biomedicines-12-01874-s001.zip › Supplementary Figure/Figure S4/Singe SNP plot of MR analysis for TL to ubm-b-1520.pdf]

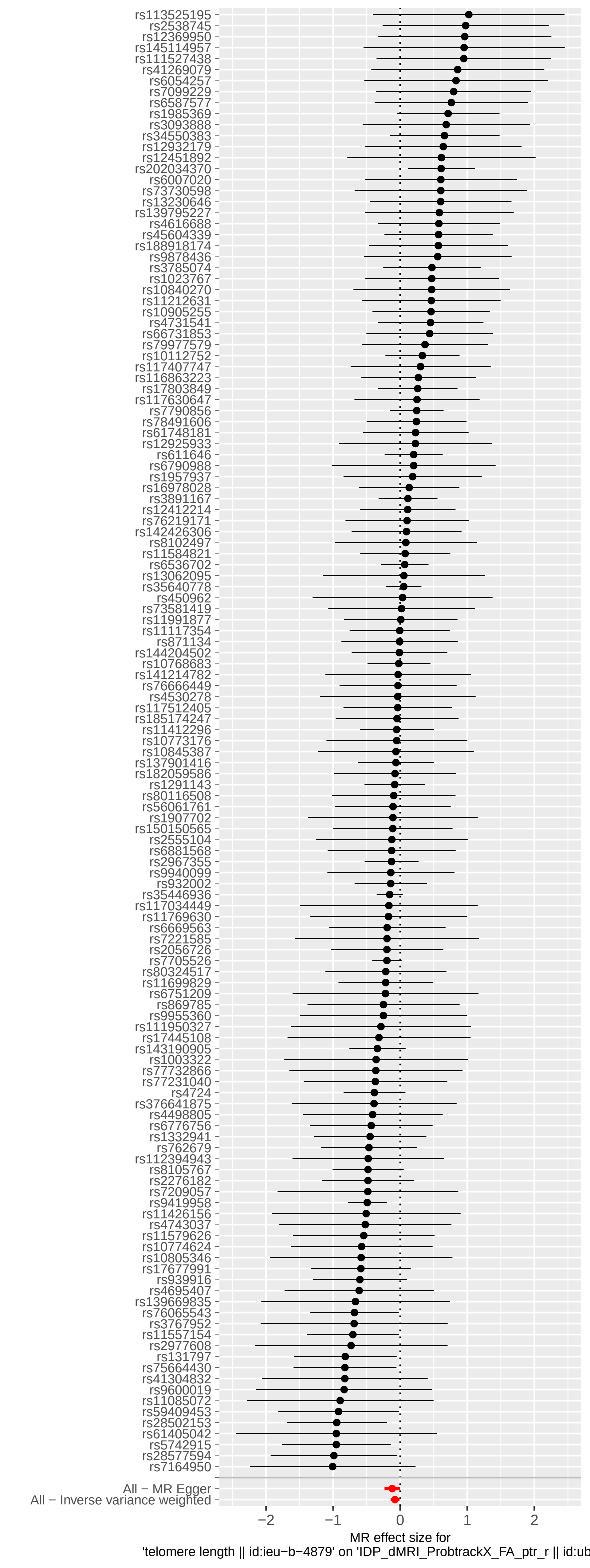
rs113525195
rs2538745
rs12369950
rs145114957
rs111527438
rs41269079
rs6054257
rs7099229
rs6587577
rs1985369
rs3093888
rs34550383
rs12932179
rs12451892
rs202034370
rs6007020
rs73730598
rs13230646
rs139795227
rs4616688
rs45604339
rs188918174
rs9878436
rs3785074
rs1023767
rs10840270
rs11212631
rs10905255
rs4731541
rs66731853
rs79977579
rs10112752
rs117407747
rs116863223
rs17803849
rs117630647
rs7790856
rs78491606
rs61748181
rs12925933
rs611646
rs6790988
rs1957937
rs16978028
rs3891167
rs12412214
rs76219171
rs142426306
rs8102497
rs11584821
rs6536702
rs13062095
rs35640778
rs450962
rs73581419
rs11991877
rs11117354
rs871134
rs144204502
rs10768683
rs141214782
rs76666449
rs4530278
rs117512405
rs185174247
rs11412296
rs10773176
rs10845387
rs137901416
rs182059586
rs1291143
rs80116508
rs56061761
rs1907702
rs150150565
rs2555104
rs6881568
rs2967355
rs9940099
rs932002
rs35446936
rs117034449
rs11769630
rs6669563
rs7221585
rs2056726
rs7705526
rs80324517
rs11699829
rs6751209
rs869785
rs9955360
rs111950327
rs17445108
rs143190905
rs1003322
rs77732866
rs77231040
rs4724
rs376641875
rs4498805
rs6776756
rs1332941
rs762679
rs112394943
rs8105767
rs2276182
rs7209057
rs9419958
rs11426156
rs4743037
rs11579626
rs10774624
rs10805346
rs17677991
rs939916
rs4695407
rs139669835
rs76065543
rs3767952
rs11557154
rs2977608
rs131797
rs75664430
rs41304832
rs9600019
rs11085072
rs59409453
rs28502153
rs61405042
rs5742915
rs28577594
rs7164950
All − MR Egger
All − Inverse variance weighted
−2
−1
0
1
2
MR effect size for
'telomere length || id:ieu−b−4879' on 'IDP_dMRI_ProbtrackX_FA_ptr_r || id:ub
